# Predictive Modeling of Drug Product Stability in Pharmaceutical Blister Packs

**DOI:** 10.3390/pharmaceutics17091233

**Published:** 2025-09-22

**Authors:** Jan Pech, Christoph Kaminski, Matthias Markus, Werner Hoheisel, Roman Heumann, Judith Winck, Markus Thommes

**Affiliations:** 1 Laboratory of Solids Process Engineering, Department of Biochemical and Chemical Engineering, Technical University Dortmund, Emil-Figge-Str. 68, 44227 Dortmund, Germany; jan.pech@tu-dortmund.de (J.P.); judith.winck@tu-dortmund.de (J.W.); 2 INVITE GmbH, Otto-Bayer-Str. 32, 51061 Cologne, Germany; heumann@invite-research.com; 3 Bayer Consumer Care AG, Peter Merian-Str. 84, 4052 Basel, Switzerland; christoph.kaminski@bayer.com; 4 Bayer AG, Kaiser-Wilhelm-Allee 1, 51373 Leverkusen, Germany; matthias.markus@bayer.com (M.M.); werner.hoheisel@bayer.com (W.H.)

**Keywords:** blister packaging, modeling, stability prediction, drug, stability, sustainability

## Abstract

**Background/Objectives:** The principal function of pharmaceutical blister packaging is to provide protection for the drug product. Moisture is regarded as a critical factor in the physical and chemical aging of drug products. The present work proposes a modeling framework to predict the performance of tablet blister materials based on the moisture uptake profile of the drug product as well as degradation characteristics of the drug substance, while the consumption of water due to degradation is included. **Methods:** The model incorporates three kinetic superimposed processes that define moisture uptake and drug stability. The processes of permeation, sorption and degradation are each described with a rate constant. Based on a mass balance, these rate processes are interconnected and the relative humidity in the blister cavity is predicted. **Results:** In a case study, the model was applied to demonstrate the feasibility of predicting the stability of blistered tablets. By establishing a correlation between the moisture uptake of the tablet and the drug stability demonstrated in the model, it was feasible to predict the drug content over shelf life. **Conclusions:** Modeling of the drug stability of blister-packed products enables a rational packaging which offers novel possibilities for reducing material in order to avoid overpackaging of pharmaceutical products. As some of the commonly used barrier materials are considered to not be sustainable, this model can be used to consider a rationally justified reduction or even abandonment of the barrier materials.

## 1. Introduction

The European Green Deal aims at achieving climate neutrality across the European continent by the year 2050 [1]. A key component of this policy initiative entails the establishment of ambitious targets for the reduction in packaging waste and the prevention of overpackaging of products. Moreover, a design-for-recycling principle is introduced to improve the suitability of packaging for circular economy [2].

A primary function of blister packaging is the protection of the drug product, thereby ensuring the quality of pharmaceutical products over their shelf life. Moisture is widely regarded as a critical factor contributing to physical and chemical aging [3,4,5]. Consequently, an adequate moisture barrier is of paramount importance in the development of blister packaging [6].

In the context of the circular economy, a preferred solution is a blister made from a single-component material [7,8]. Due to the absence of high barrier layers in single-component polymeric packaging materials, the simultaneous assurance of pharmaceutical quality and recyclability represents a key challenge in the development of sustainable blister packaging [9,10].

The selection of blister packs typically involves an iterative process, whereby stability studies are often conducted following a trial-and-error approach. This procedure offers potential for economic and efficiency-driven optimization by narrowing down suitable packaging candidates, complementing but not replacing regulatory.

Based on the interdependency of moisture content and drug stability, several model approaches for the prediction of moisture uptake were developed, considering moisture uptake as a surrogate parameter for predicting shelf life. Numerical approaches were utilized to approximate the relative humidity and product moisture contents in packages as a function of time [11,12,13,14]. Chen and Li published an analytical solution for predicting the moisture uptake by packaged products [15]. Within these approaches, the kinetics of water permeation into the packaging and the sorption isotherm of the drug product are interconnected, whereby a maintained equilibrium state inside the package is assumed. Furthermore, moisture in the headspace volume of the packaging is neglected. A more general approach was established by Remmelgas et al. through the integration of the sorption kinetics of the drug product and the water vapor in the headspace into their modeling framework [16,17]. The humidity-corrected Arrhenius approach was employed to establish a correlation between the degradation rate and the relative humidity in the environment of the tablet [18,19]. This allowed a mechanistic prediction of the drug content over the shelf life by considering the products moisture uptake profile in packaging [20,21].

However, none of the previous approaches take into account the mass of water used for degradation within a holistic approach. This may result in an inaccurate estimation of the moisture content, leading to a systematic deviation in the predicted drug content. Furthermore, the model approach used is sometimes not clear, and the algorithms are usually not disclosed. This reduces scientific value and avoids any further development of these software applications by the scientific community.

The aim of the study was to establish a modeling framework to predict the performance of tablet blister materials considering various environmental conditions as well as degradation characteristics of the drug substance, explicitly accounting for the consumption of water associated with hydrolytic degradation of the drug. The chemical stability of the drug was selected as a performance indicator. The concept was introduced at the IAPRI Conference in 2024 and intensively discussed by experts in the field [22]. In the pursuit of developing a comprehensive modeling framework that can be universally applied to a wide variety of packaging product combinations, it is imperative to consider the amount of water consumed by the degradation process.

The selection of packaging is based on the barrier properties of the packaging material and the material properties of the formulation with regard to sorption and degradation. This enables a rational selection of packaging material, which appears superior to iterative methodology in terms of costs and efficiency. Aligning with the quality-by-design paradigm, the modeling framework facilitates the identification of crucial inquiries in packaging development concerning material selection and packaging design. This approach offers the additional advantage of enhancing the sustainability of pharmaceutical packaging by preventing the unjustified use of unsustainable barrier layers and avoiding overpackaging.

## 2. Results and Discussion

### 2.1. Model Design

**Figure 1:**
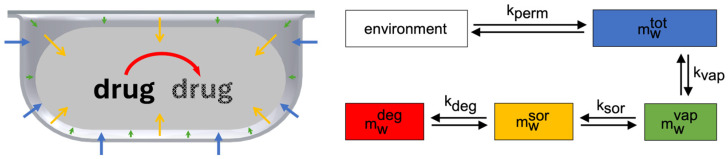
Schematic illustration of the occurring mass flows of water permeated into cavity (blue arrows), water vapor in cavity (green arrows), sorption of water to tablet (yellow arrows) and consumption of water due to degradation of the drug substance to degradation products (shaded drug) (red arrow).

Water ingressing into a tablet blister experiences different stages, which results from the encapsulation of water transport processes within the blister. Hence, the tablet blister can be regarded as a multi-compartment system in which the water can be located in different compartments (Figure 1). Transfer of water is only possible between adjacent compartments. First, some water molecules will appear in the gas phase ${(m}_{w}^{\mathrm{vap}})$, other molecules will be sorbed to the tablet $\left(m_{w}^{\mathrm{sor}}\right)$, and then, water molecules are available for decomposition of the drug contained in the tablet ($m_{w}^{\deg})$.

The total mass within the blister ($m_{w}^{\mathrm{tot}}$) is invariably the sum of these individual contributions:

(1) $$m_{w}^{\mathrm{tot}}=m_{w}^{\mathrm{vap}}+m_{w}^{\mathrm{sor}}+m_{w}^{\deg}$$

Moreover, the transport of water into the cavity, water sorption and degradation are rate processes, thus the quantity of water in each term changes over time. Since these rate processes are coupled, the quantity of water in the gas phase is usually altered with time. In a blister system that is not in equilibrium, the mass balance is applicable to each time step and is therefore time dependent.

(2) $$m_{w,t}^{\mathrm{vap}}=m_{w,t}^{\mathrm{tot}}-m_{w,t}^{\mathrm{sor}}-m_{w,t}^{\deg}$$

$m_{w,t}^{\mathrm{tot}}$, $m_{w,t}^{\mathrm{sor}}$ and $m_{w,t}^{\deg}$ are described individually and can be analytically accessed. $m_{w,t}^{\mathrm{vap}}$ results from the mass balance and is directly related to the relative humidity in the blister cavity. After calculation of all individual sub-processes, Equation (2) is solved with respect to time.

The mass of water within the system at a time t ($m_{w,t}^{\mathrm{tot}}$) is composed of two constituent components: the initial mass of water within the system, prior to the commencement of the time interval under consideration, and the additional water mass that is introduced into the cavity during the specified time interval. The mass of water that has permeated the cavity is thus:

(3) $$m_{w,t}^{\mathrm{vap}}\;=\;m_{w,t-1}^{\mathrm{tot}}\;+\;\int_{t-1}^{t}\frac{dm_{w,t}^{\mathrm{tot}}}{\mathrm{dt}}\mathrm{dt}\;-{\;m}_{w,t}^{\mathrm{sor}}\;-\;m_{w,t}^{\deg}$$

Equation (1) can be applied and integrated into Equation (3):

(4) $$m_{w,t}^{\mathrm{vap}}\;=\;m_{w,t-1}^{\mathrm{vap}}+m_{w,t-1}^{\mathrm{sor}}\;+\;m_{w,t-1}^{\deg}\;+\;\int_{t-1}^{t}\frac{dm_{w,t}^{\mathrm{tot}}}{\mathrm{dt}}\mathrm{dt}\;-\;m_{w,t}^{\mathrm{sor}}\;-\;m_{w,t}^{\deg}$$

$m_{w,t-1}^{\mathrm{vap}}$ and $\int_{t-1}^{t}\frac{dm_{w,t}^{\mathrm{tot}}}{\mathrm{dt}}\mathrm{dt}$ describe the mass in the gas phase of the cavity if there is no subsequent sorption by the tablet and degradation in the tablet. As degradation and sorption alter the water mass in the gas phase, a coupling process occurs via this mechanism. Consequently, the three kinetic processes are interconnected by the mass of water present in the gas phase of the cavity. This directly impacts the water mass that permeates the system by influencing the concentration gradient, necessitating the implementation of an iterative solution. The convolution of the sub-processes converges but does not necessarily describe a state of equilibrium. The relative humidity is determined from the mass of water in the gas phase and converges to a value over the iterations.

All sub-processes follow a first-order kinetic if they run independently of each other. Permeation, sorption and degradation have analytical solutions that commonly obey the following pattern:

(5) $$\frac{dm_{w}}{\mathrm{dt}}\;=\;-k\cdot (m_{w,\infty }-m_{w})$$

The upper limit of function is referred to as $m_{w,\infty }$. Within the context of permeation and sorption, this is defined as equilibrium at a specific relative humidity and temperature. In the context of degradation, it would be the quantity of water required to degrade the entire drug contained in the drug product. k represents the rate constant of the respective sub-process.

Integration of Equation (5) over a fixed time interval enables the determination of the change in water mass in a compartment over a time step ∆t:

(6) $$\frac{1}{m_{w,\infty }\;-\;m_{w}}\int_{m_{w,t-1}}^{m_{w,t}}\mathrm{dm}\;=\;-k\int_{t-1}^{t}\mathrm{dt}$$

(7) $${∆m}_{w,∆t}=m_{w,\infty }-{(m}_{w,\infty }-m_{w,t-1})\cdot e^{-k\cdot ∆t}$$

### 2.2. Model Parametrization

#### 2.2.1. General

Each mathematical model will require specific parameters, which are commonly acquired for a certain application. Specifically, the permeation, sorption as well as degradation processes must be characterized by appropriate parameters. However, this work is dedicated to model development and utilizes literature data exclusively for the purpose of parameterization. Moreover, the constants employed in this study are given in Table 1.

#### 2.2.2. Permeation

The permeation process into the blister cavity is characterized by two fundamental parameters, namely the **equilibrium mass of water in the vapor phase of the blister cavity (**$m_{w,\infty }^{\mathrm{vap}}$**)** and the rate constant of permeation (k_perm_). The equilibrium mass depends on the relative humidity in the environment (outside of the blister) as well as the volume of the blister cavity ($V^{\mathrm{vap}}$). On the other hand, the rate constant is a property of the blister material which can be ascertained within permeation tests.

The driving force of permeation is the difference in the partial vapor pressures of the water between the interior of the blister cavity ($p_{w}^{\mathrm{vap}}$) and its environment outside ($p_{w}^{\mathrm{env}}$), and can thus be expressed as relative humidity ($\varphi_{w}$) by normalizing to the saturation vapor pressure ($p_{w}^{\mathrm{LV}}$).

(8) $$\varphi_{w}=\frac{p_{w}}{p_{w}^{\mathrm{LV}}}$$

The vapor pressure of water is proportional to the mass of water in the gas phase of the blister cavity ($m_{w}^{\mathrm{vap}}$), as stipulated by the ideal gas law.

(9) $$m_{w}^{\mathrm{vap}}=\frac{\varphi_{w}^{\mathrm{vap}}\cdot M_{w}\cdot V^{\mathrm{vap}}\cdot p_{w}^{\mathrm{LV}}}{R\cdot T}$$

The equilibrium of the mass of water in the gas phase of the blister cavity is determined by the insertion of the relative humidity of the environment. Therefore, $m_{w,\infty }^{\mathrm{vap}}$ is contingent on the relative humidity outside of the blister.

(10) $$m_{w,\infty }^{\mathrm{vap}}=\frac{\varphi_{w}^{\mathrm{env}}\cdot M_{w}\cdot V^{\mathrm{vap}}\cdot p_{w}^{\mathrm{LV}}}{R\cdot T}$$

The permeation of water vapor through a plane sheet can be described by a first-order kinetic, characterized by the **rate constant of permeation (**$k_{\mathrm{perm}}$**)** [27]. This concept can be transferred to the mass flow of water into the blister cavity:

(11) $$\frac{dm_{w}^{\mathrm{vap}}}{\mathrm{dt}}\;=\;k_{\mathrm{perm}}\cdot \left(m_{w,\infty }^{\mathrm{vap}}-\;m_{w}^{\mathrm{vap}}\right)$$

**Figure 2:**
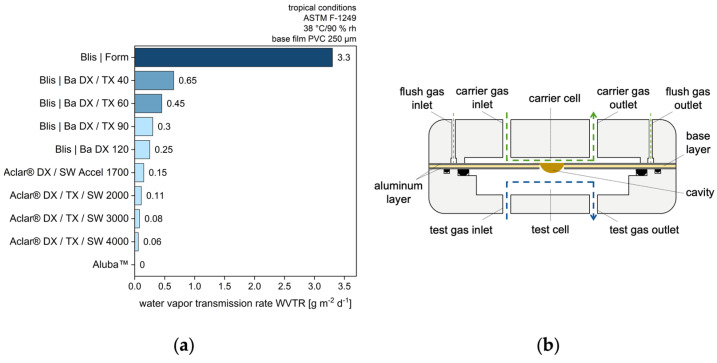
Permeation characterization of blister films and cavities. (**a**) Water vapor transmission rate (WVTR) for pristine blister foils according to [30]; (**b**) Experimental setup of permeation tester examining a blister cavity according to [31].

This rate constant is derived from the experimentally determined water barrier properties, like the water vapor transmission rate (WVTR). This is defined as the mass flow of water vapor passing through a given surface under certain conditions of temperature and relative humidity. For pristine blister foils, it is specified by the foil manufacturer (Figure 2, left). Furthermore, permeation tests (Figure 2, right) are widely employed for experimental determination of the WVTR, allowing the measurement of processed blister foils such as thermoformed blister cavities [28,29]. In these tests, the donor cell ($m_{w,\infty }^{\mathrm{wet}}$) and the acceptor cell ($m_{w}^{\mathrm{dry}}$) are separated by the blister cavity, while the water vapor flow is measured (Figure 2, right).

In steady state of the WVTR measurement, the following applies, where $A^{\mathrm{blis}}$ is defined as the surface area of the blister cavity:

(12) $$\mathrm{WVTR}\cdot A^{\mathrm{blis}}\;={\;k}_{\mathrm{perm}}\cdot \left(m_{w,\infty }^{\mathrm{wet}}\;-\;m_{w}^{\mathrm{dry}}\right)$$

Water vapor that has permeated through the foil is removed directly in the acceptor compartment ($m_{w}^{\mathrm{dry}}\;=\;0$), thus acting as a sink.

(13) $$\mathrm{WVTR}\cdot A^{\mathrm{blis}}=k_{\mathrm{perm}}\cdot m_{w,\infty }^{\mathrm{wet}}$$

The relative humidity is established within the cell situated in front of the sample (i.e., the donor compartment), which is proportional to the mass contained within the gas phase (Equation (10)).

(14) $$\mathrm{WVTR}\cdot A^{\mathrm{blis}}=k_{\mathrm{perm}}\cdot \frac{\varphi_{w}^{\mathrm{wet}}\cdot M_{w}\cdot V^{\mathrm{vap}}\cdot p_{w}^{\mathrm{LV}}}{R\cdot T}$$

This is rearranged in order to obtain the rate constant of permeation:

(15) $$k_{\mathrm{perm}}\;=\;\frac{\mathrm{WVTR}\cdot A^{\mathrm{blis}}\cdot R\cdot T}{\varphi_{w}^{\mathrm{wet}}\cdot M_{w}\cdot V^{\mathrm{vap}}\cdot p_{w}^{\mathrm{LV}}}$$

The permeation can be defined by the upper limit, which is related to the relative humidity in the environment of the blister, and a characteristic rate constant, which quantifies the water barrier of the blister.

#### 2.2.3. Sorption

The sorption process can be described by the **mass of water sorbed to tablet at equilibrium (**$m_{w,\infty }^{\mathrm{sor}}$**)** and the rate constant of sorption ($k_{\mathrm{sorp}}$). The equilibrium mass depends on the relative humidity in the direct environment of the tablet (inside of the blister). In contrast, the rate constant of sorption is a material property of the tablet and can be determined within sorption testing.

**Figure 3:**
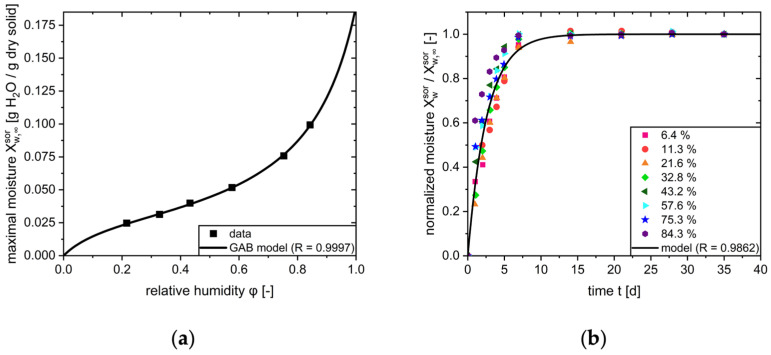
Sorption properties of a tablet formulation at 25 °C in accordance with [15]. (**a**) Sorption isotherm after application of the GAB model; (**b**) Normalized sorption kinetics at different relative humidities.

The driving force of sorption is defined as the difference between the mass of water already sorbed by the tablet ($m_{w,t}^{\mathrm{sor}}$) and the sorbed mass of water at equilibrium ($m_{w,\infty }^{\mathrm{sor}}$). This equilibrium results from the material properties of the tablet components and is derived directly from the sorption isotherm. The Guggenheimer–Anderson–De Boer (GAB) model represents a widely applicable framework for modeling the experimentally determined sorption behavior of a large number of different solid-state systems [32,33,34]. However, it is also possible to utilize alternative models. By adjusting the model parameters ($w_{m}$, $C_{\mathrm{GAB}}$, $K_{\mathrm{GAB}}$) a mathematical description the moisture of the sorption isotherm can be obtained (Figure 3, left):

(16) $$X_{w,\infty }^{\mathrm{sor}}\;=\;\frac{m_{w,\infty }^{\mathrm{sor}}}{m_{\mathrm{dry}}^{\mathrm{tab}}}\;=\;w_{m}\cdot \frac{C_{\mathrm{GAB}}\cdot K_{\mathrm{GAB}}\cdot \varphi_{w}^{\mathrm{vap}}}{\left(1\;-K_{\mathrm{GAB}}\cdot \varphi_{w}^{\mathrm{vap}}\right)\cdot \left(1\;-K_{\mathrm{GAB}}\cdot \varphi_{w}^{\mathrm{vap}}\;+\;{C_{\mathrm{GAB}}\cdot K}_{\mathrm{GAB}}\cdot \varphi_{w}^{\mathrm{vap}}\right)}$$

Since the dry mass of the solid ($m_{\mathrm{dry}}^{\mathrm{tab}}$) is a constant quantity, rearranging the GAB equation enables calculation of the total mass of sorbed water ($m_{w,\infty }^{\mathrm{sor}}$) from the equilibrium moisture load ($X_{w,\infty }^{\mathrm{sor}}$) at a given relative humidity:

(17) $$m_{w,\infty }^{\mathrm{sor}}\;=\;w_{m}\cdot \frac{C_{\mathrm{GAB}}\cdot K_{\mathrm{GAB}}\cdot \varphi_{w}^{\mathrm{vap}}}{\left(1\;-\;K_{\mathrm{GAB}}\cdot \varphi_{w}^{\mathrm{vap}}\right)\cdot \left(1\;-K_{\mathrm{GAB}}\cdot \varphi_{w}^{\mathrm{vap}}\;+\;{C_{\mathrm{GAB}}\cdot K}_{\mathrm{GAB}}\cdot \varphi_{w}^{\mathrm{vap}}\right)}\cdot m_{\mathrm{dry}}^{\mathrm{tab}}$$

Therefore, $m_{w,\infty }^{\mathrm{sor}}$ is contingent on the relative humidity in the blister cavity.

The sorption of water to the tablet follows a first-order kinetic, characterized by the **rate constant of sorption (**$k_{\mathrm{sor}}$**)**:

(18) $$\frac{dm_{w}^{\mathrm{sor}}}{\mathrm{dt}}\;=\;k_{\mathrm{sor}}\cdot \left(m_{w,\infty }^{\mathrm{sor}}-\;m_{w}^{\mathrm{sor}}\right)$$

This rate constant can be determined by analyzing the time-dependent moisture uptake of the tablet at a certain relative humidity level. Dynamic vapor sorption (DVS) analyses are widely used to determine the sorption isotherm and sorption kinetics. In these experiments, the increase in mass of a dry tablet is measured in response to a set relative humidity within the tablet environment.

By normalizing the moisture load ($X_{w}^{\mathrm{sor}}$) to the equilibrium moisture load of the set humidity level ($X_{w,\infty }^{\mathrm{sor}}$), a single rate constant for all humidity levels can be determined (Figure 3, right):

(19) $$\frac{X_{w}^{\mathrm{sor}}}{X_{w,\infty }^{\mathrm{sor}}}\;=\;1\;-{\;e}^{-k_{\mathrm{sor}}\cdot t}$$

In case $k_{\mathrm{sorp}}$ changes depending on the relative humidity under consideration, a function of $k_{\mathrm{sor}}(\varphi_{w})$ can also be utilized. The sorption can be characterized by the upper limit, which denotes the mass of water sorbed to the tablet in equilibrium for a given ambient humidity of the tablet. Additionally, the sorption constant describes the kinetic rate of the sorption process.

#### 2.2.4. Degradation

**Figure 4:**
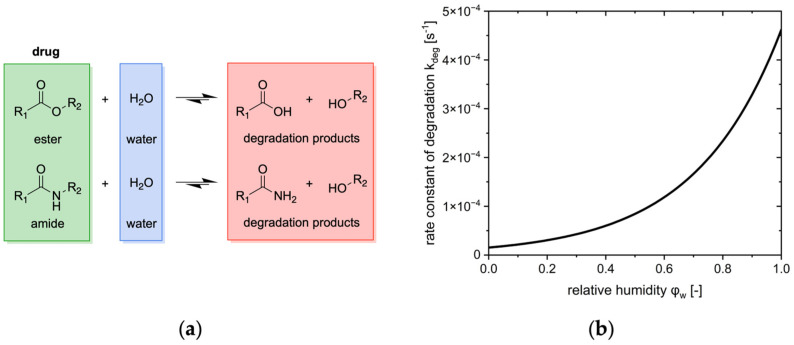
Degradation behavior of the contained drug. (**a**) Degradation reactions of hydrolysis-sensitive drugs; (**b**) Degradation rate constant of cilazapril at different relative humidities and 40 °C in accordance with [26].

Given the presence of moisture-sensitive bonds (e.g., ester or amide bonds) in many drug substances, water is assumed to be a reactant in degradation reactions, such as hydrolysis (Figure 4, left). However, it is acknowledged that degradation reactions can occur in the absence of moisture.

The degradation process can be fully delineated by the **mass of water required to degrade the mass of drug contained in the tablet (**$m_{w,\infty }^{\deg}$**)** and the degradation rate constant ($k_{\deg}$).

The upper limit depends solely on the drug load of the tablet. Conversely, the degradation rate constant is contingent on the environmental conditions of the tablet, namely temperature and relative humidity.

The driving force of degradation is characterized by the difference between the mass of water required to degrade the entire drug contained ($m_{w,\infty }^{\deg}$) and the mass of water already consumed for degradation ($m_{w}^{\deg}$). For water-dependent degradation reactions, the mass of water consumed to degradation is directly proportional to the mass of drug degraded. The stoichiometry of this reaction is considered by the stoichiometric factor ($\nu_{\deg}$). The mass of water required for the degradation process can then be calculated, given the molar masses of the drug ($M_{d}$) and water ($M_{w}$):

(20) $$m_{w}^{\deg}\;=\;\nu_{\deg}\cdot \frac{M_{w}}{M_{d}}\cdot m_{d}^{\deg}$$

By inserting the total mass of drug contained in the tablet, the mass of water required to degrade the entire drug can be calculated.

(21) $$m_{w,\infty }^{\deg}=\nu_{\deg}\cdot \frac{M_{w}}{M_{d}}\cdot m_{d,\infty }^{\deg}$$

Since the stoichiometry of the reaction and the molar masses remain constant over time, the upper limit of the degradation is designated as a constant.

The degradation kinetic of the drug contained within the tablet, as well as the consumption of water within this reaction, can often be described by a first-order kinetic, defined by the **degradation rate constant (**$k_{\deg}$**)**.

(22) $$\frac{dm_{w}^{\deg}}{\mathrm{dt}}=k_{\deg}\cdot \left(m_{w,\infty }^{\deg}-\;m_{w}^{\deg}\right)$$

This rate constant is typically determined in the context of stability studies, wherein the drug content is monitored over time. Given that the degradation kinetic is contingent upon the environmental conditions of the tablet, stability studies must be conducted under variations in these parameters. Through the exposition of tablets to different relative humidities, the degradation rate constant can be determined for different moisture contents. It is assumed that the tablets possess an equilibrium moisture content and the relative humidity in the environment is consistent with the moisture content of the tablets.

A humidity-corrected Arrhenius approach is a widely established model that relates the rate constant of degradation directly to the various environmental conditions of the tablets, i.e., temperature (T) and relative humidity ($\varphi_{w}^{\mathrm{vap}}$) [18,35,36].

(23) $$k_{\deg}=k_{\deg,0}\cdot \exp\left(\frac{E_{a}}{R\cdot T}\right)\cdot \exp\left(B\cdot \varphi_{w}^{\mathrm{vap}}\right)$$

The temperature-dependency is assessed by adjusting the pre-exponential factor ($k_{\deg,0}$) and the activation energy ($E_{a}$). The influence of the relative humidity on the reaction rate is determined by the empirical humidity sensitivity factor (B). This parameter is derived from the experimentally determined rate constants at varying relative humidity levels. For isothermal conditions, the degradation constant is a function of the relative humidity in the tablet’s environment (Figure 4, right). In the microclimate surrounding a drug particle situated within a tablet, the moisture content of the excipients may contribute to the availability of moisture for the reaction [37,38]. As the moisture load of the tablet corresponds to its water activity, this governs the availability of water for degradation.

The degradation process can be modeled by determining the upper limit, which represents the mass of water required to degrade the entire drug, and the rate constant at which the reaction occurs.

### 2.3. Model Implementation

**Figure 5:**
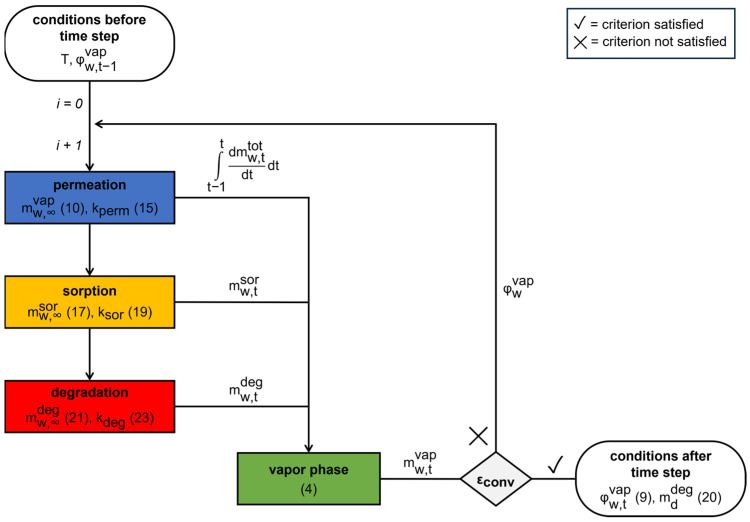
Flow chart illustrating the calculation algorithm for a single time step for numerous iterations (i). Numbers in brackets refer to the respective equations.

In order to utilize the proposed model, it is necessary to examine the permeation, sorption and degradation kinetics, as well as their interactions. Hence, Equations (11), (18) and (22) must be solved simultaneously and coupled within Equation (2). Due to the high interdependency of these, a numerical approach was employed, whereby the individual kinetics were analytically solved for discrete time points, and the mass balance was iteratively solved (Figure 5).

All individual sub-processes are calculated for discrete time steps and Equation (4) is solved until a convergence criterion is reached: After the calculation of the permeated water, the mass of water sorbed to the tablet is determined. Subsequent to the incorporation of the water into the tablet, its removal from the gas phase occurs. The water used for degradation at the resulting relative humidity in the blister cavity is ascertained and considered within the mass balance. The next iteration step is carried out based on the relative humidity in the cavity phase that changed within the iteration step.

The model was implemented in Python (version 3.9.6, Python Software Foundation, Delaware, DE, USA) as open-source programming language.

The time step size and convergence limit are relevant discretization parameters, which define the accuracy of the calculations and the computational effort. The selection of a smaller time step size results in enhanced accuracy, whilst concomitantly incurring extended computational times. Conversely, a large time step size can lead to systematic deviations, whereas a reduction in the computation time can be achieved. The selection of the time step size is contingent on the fastest sub-process indicated by the highest rate constant. In order to provide a parameter-independent implementation, a dynamic time step selection based on a **time step size selection criterion (**$\sigma_{\Delta t}$**)** is required, whereby this is based on the highest rate constant (Equation (24)). Within the context of Equation (7), this corresponds to the relative approximation to the equilibrium state for the fastest sub-process. This equation is derived directly from the definition of the process as a first-order rate process.

(24) $$\Delta t=\frac{\ln\left(1-\sigma_{\Delta t}\right)}{{-k}_{\mathrm{fast}}}$$

For a criterion of ${\sigma_{\Delta t}\;=\;10}^{-2}$, Δt refers to the time step size to reach 1% of the equilibrium of the rate process under consideration. Since only a small fraction of the equilibrium is reached with the selected time step size Δt, fast processes can also be integrated in a numerically stable manner.

**Figure 6:**
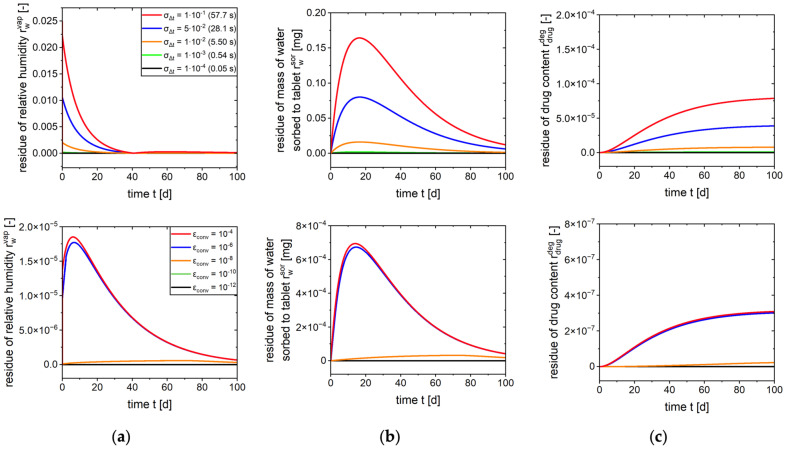
Sensitivity analysis regarding time step size (upper row, time step size in brackets) and convergence criterion (lower row) on predicted (**a**) cavity humidity, (**b**) mass of water sorbed to tablet (**b**,**c**) drug content over time.

A sensitivity analysis was conducted, in which the temporal progression of residuals of coarser time step sizes to a reference solution computed with a particularly small time step size was analyzed considering permeation, sorption and degradation (Figure 6, upper row). Following this, a time step selection criterion was considered to be sufficient. Utilizing the time step size criterion, the time step size can be dynamically adjusted at the beginning of each time step during the calculation process. This ensures numerical stability when rate constants are altered during the calculation.

Since the mass balance is solved iteratively, the mass balance converges to an exact solution as the iterations progress. Consequently, a second criterion is introduced to ensure a sufficient degree of convergence. Equation (2) is solved iteratively for each time step until a **convergence limit (**$\epsilon_{\mathrm{conv}}$**)** is met, which describes the permissible relative deviation of the calculated water masses from the previous iteration step. In the event that the deviation from the previous iteration step is no greater than this limit, the mass balance is considered to be solved, and thus the subsequent calculation of the next time step is initiated.

A sensitivity analysis with regard to the convergence limit was performed using a dynamic time step size selection (${\sigma_{\Delta t}=10}^{-2}$). Considering the residues for the three sub-processes, a limit of $\epsilon_{\mathrm{conv}}\;=\;10^{-8}$ was found to be adequate (Figure 6, lower row). Since the convergence limit is rather independent of the specified parameters, this is taken to be universally applicable.

### 2.4. Model Application

The model is based on the following assumptions:

- The barrier properties of the blister, expressed as the rate constant of permeation, are determined in the steady state of water permeation. The saturation of the blister film is neglected with respect to the multi-year time scale of the simulation.
- All rate constants of the different sub-processes are assumed as constants within a time step.
- The sorption properties of the formulation remain constant over the simulation period. As degradation progresses, the composition of the tablet is assumed not be altered due to the accumulation of the degradation product. This may lead to deviations from the originally determined sorption rate constant and sorption isotherm.

In the context of a case study, the model was applied to demonstrate the feasibility of predicting the stability of blistered tablets. As the objective of this case study is not to simulate the stability of a real formulation, it is instead intended to provide insight into the functionality of the model using the example of the moisture-sensitive drug cilazapril. For this set of input data, the information provided by the simulations is less valuable due to the combination of sorption data from a placebo tablet with the degradation properties in another substance system. Moreover, sorption properties at 25 °C are included in the simulation due to the lack of sorption data at 40 °C, and hence there is no combination of isothermal transport processes. However, the temperature only has an influence on magnitude of the parameter. All underlying models remain applicable.

**Figure 7:**
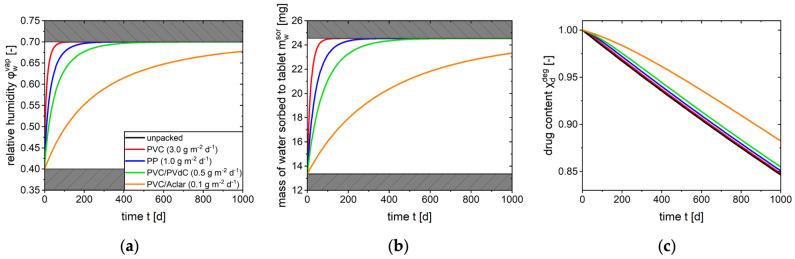
Simulation results of case study. Prediction of (**a**) relative humidity in cavity, (**b**) moisture uptake of tablet and (**c**) drug content over time at 40 °C and 70% assuming an initial relative humidity of 40% in blister cavity.

An initial relative humidity of 40% in the gas phase of the blister cavity and an ambient air humidity of 70% were assumed. For identical tablets, the relative humidity in the blister cavity (Figure 7a) and the moisture increase in the tablet (Figure 7b) were predicted in various blister materials with a thickness of 100 µm after thermoforming. Based on the correlation between the moisture uptake of the tablet and the drug stability shown in the model, it was possible to predict the drug content over a period of about 3 years (Figure 7c).

As a result of the difference in relative humidity between the environment and the cavity, moisture enters the blister. Subsequent to the increase in relative humidity, the tablet begins the absorption of moisture (Figure 1). A higher barrier effect (e.g., PVC/Aclar) results in slower water uptake by the tablet. However, only a high barrier leads to a visible slowdown in moisture uptake in the system under consideration.

A direct correlation is also observed between the barrier properties of the packaging material and the predicted stability of the drug. The degradation of the drug is found to be slower in blisters with a higher barrier. Since the moisture uptake and the increase in relative humidity in the blister cavity are retarded in high barrier materials, this is especially evident in the initial period of shelf life. Nevertheless, drug content differs only by approximately 3% compared to an unpacked tablet after 2 years of storage.

Furthermore, the drug content over time with a low barrier differs only slightly from the drug content-time profile of an unpacked tablet. Thus, with a low barrier (e.g., PVC), there is no substantial slowdown in degradation for this particular drug formulation. In this case, a high barrier is required as packaging materials with a low barrier do not offer sufficient protection against moisture and do not contribute to preserving quality.

The results of this simulation provide a rational basis for selecting potential packaging materials for further stability tests. Furthermore, a substantial reduction in experimental costs can be achieved by utilizing a mechanistic model compared to the subsequent conduction of stability tests within an iterative packaging selection process.

## 3. Conclusions

In this work, a novel approach for predicting the moisture uptake and the drug content of a blister-packed tablet was presented. This approach is based on the kinetics of the interconnected transport processes of water. For the purpose of this study, a detailed mathematical description of water ingress into the blister cavity, moisture uptake and water used for degradation was provided. To this end, the rate constants of the respective transport processes were derived. Since the modeling framework incorporates the consumption of water due to degradation, this represents a holistic approach to predict the drug stability of blister-packed pharmaceutical products.

The implementation of the modeling framework involved the application of an iterative solving algorithm for the interconnection of the rate-processes of permeation, sorption and degradation. The parameters found to be relevant in the context of the implementation (time step size selection criterion $\sigma_{\Delta t}$, convergence limit $\epsilon_{\mathrm{conv}}$) were defined and their influence on the modeling result was investigated.

In order to provide insights into the model application, a case study based on literature data was conducted. The case study elucidated the effect of the barrier properties of the blister on the moisture uptake and the drug content over shelf life. As the drug content over shelf life in a low-barrier blister was found to be comparable to that of an unpacked tablet, a low-barrier blister is not providing any protection for the product under consideration. In contrast, a substantial improvement in drug stability was achieved in packaging materials with a higher barrier effect.

Predictive modeling of drug product stability in pharmaceutical blister packs enables a rational selection of packaging materials, as the properties of the individual product are directly correlated with the barrier properties of the materials used. This allows for greater efficiency in addressing key questions within pharmaceutical product development. By following the approach of quality-by-design, it opens new possibilities for reducing the barrier levels, thus avoiding the overpackaging of pharmaceutical products. Since many of the barrier layers used severely limit the recyclability of pharmaceutical blister packs, avoiding these layers serves to increase the sustainability.

## Figures and Tables

**Table 1:** Parameters used for modeling. The blister cavity is geometrically adapted to the tablet.

Parameter	Symbol	Value	Unit	Reference
*Permeation*				
Saturation pressure at 40 °C and 100 kPa	$p_{w}^{\mathrm{LV}}$	7.39	kPa	[23]
Molecular mass of water	$M_{w}$	18.0	g mol^−1^	-
Gas volume of blister cavity *	$V^{\mathrm{vap}}$	0.41	ml	-
Ideal gas constant	R	8.314	J mol^−1^ K^−1^	-
Temperature *	T	313	K	-
Surface area of blister cavity *	$A^{\mathrm{blis}}$	3.9	cm^2^	-
*Sorption*				
Dry mass of tablet	$m_{\mathrm{dry}}^{\mathrm{tab}}$	364	mg	[24]
GAB parameter	$w_{m}$	0.0296	-	[24]
GAB parameter	$C_{\mathrm{GAB}}$	8.93	-	[24]
GAB parameter	$K_{\mathrm{GAB}}$	0.847	-	[24]
Rate constant of sorption	$k_{\mathrm{sor}}$	4.41	s^−1^	-
*Degradation*				
Molecular mass of cilazapril	$M_{d}$	417.5	g mol^−1^	[25]
Mass of cilazapril per tablet *	$m_{d,\infty }^{\deg}$	10	mg	-
Pre-exponential factor	$k_{\deg,0}$	5.43·10^−18^	s^−1^	[26]
Activation energy	$E_{a}$	1.71·10^5^	J mol^−1^	[26]
Humidity sensitivity factor	B	3.4	-	[26]

* Parameters that are selected within commonly used ranges.

## Data Availability

Data will be made available on request.

## Abbreviations

The following abbreviations are used in this manuscript:

DVS	Dynamic vapor sorption
GAB	Guggenheim–Anderson–De Boer
PP	Polypropylene
PVC	Polyvinyl chloride
PVdC	Polyvinylidene chloride
WVTR	Water vapor transmission rate
